# Cytotoxic Effects of Compounds Isolated from *Ricinodendron heudelotii*

**DOI:** 10.3390/molecules24010145

**Published:** 2019-01-02

**Authors:** Omolara F. Yakubu, Abiodun H. Adebayo, Titilope M. Dokunmu, Ying-Jun Zhang, Emeka E.J. Iweala

**Affiliations:** 1 Department of Biochemistry, College of Science and Technology, Covenant University, Canaan Land, Ota PMB 1023, Ogun State, Nigeria; omolara.yakubu@covenantuniversity.edu.ng (O.F.Y.); titilope.dokunmu@covenantuniversity.edu.ng (T.M.D.); emeka.iweala@covenantuniversity.edu.ng (E.E.J.I.); 2 State Key Laboratory of Phytochemistry and Plant Resources of West China, Kunming Institute of Botany, Chinese Academy of Sciences. #132, Lanhei Road, Heilongtan, Kunming 650201, China

**Keywords:** anticancer, *Ricinodendron heudelotii*, chemoprevention

## Abstract

This study was designed to explore the in vitro anticancer effects of the bioactive compounds isolated from *Ricinodendron heudelotii* on selected cancer cell lines. The leaves of the plant were extracted with ethanol and partitioned in sequence with petroleum ether, ethyl acetate, and *n*-butanol. The ethyl acetate fraction was phytochemically studied using thin layer chromatography (TLC) and column chromatography (CC). Structural elucidation of pure compounds obtained from the ethyl acetate fraction was done using mass spectra, ^1^H-NMR, and ^13^C-NMR analysis. The isolated compounds were subsequently screened using five different cancer cell lines: HL-60, SMMC-7721, A-549, MCF-7, SW-480, and normal lung epithelial cell line, BEAS-2B, to assess their cytotoxic effects. Nine compounds were isolated and structurally elucidated as gallic acid, gallic acid ethyl ester, corilagin, quercetin-3-*O*-rhamnoside, myricetin-3-*O*-rhamnoside, 1,4,6-tri-*O*-galloyl glucose, 3,4,6-tri-*O*-galloyl glucose, 1,2,6-tri-*O*-galloyl glucose, and 4,6-di-*O*-galloyl glucose. Corilagin exhibited the most cytotoxic activity with an IC_50_ value of 33.18 μg/mL against MCF-7 cells, which were comparable to cisplatin with an IC_50_ value of 27.43 µg/mL. The result suggests that corilagin isolated from *R. heudelotii* has the potential to be developed as an effective therapeutic agent against the growth of breast cancer cells.

## 1. Introduction

Cancer is a major cause of death across the globe, affecting all age groups [1]. The World Health Organization has predicted that cancer cases will increase from 14 million in 2012 to 22 million in the next four decades [2]. It has caused a great sense of burden both to individual lives and the society at large [3]. The absolute cure of cancer remains a big challenge despite available anticancer agents, awareness campaigns, and prevention strategies. Breast and prostate cancer have the highest incidence in women and men, respectively [4]. It is therefore pertinent to keep exploring for novel chemotherapeutic agents that can evade drug-resistant cancer cells. Natural or natural based anti-cancer drugs, such as vincristine, paclitaxel, bleomycin, and others, have been clinically used over the years [5]. *Ricinodendron heudelotii* (family: Euphorbiceae) plant parts have been used in traditional medicine to treat cough, stomach ailments, and pain related to child birth. Decoctions and infusions are mostly prepared from the leaves and stem bark. Traditional practitioners prescribe it for women suffering from miscarriages [6]. Phytochemical screening of the leaf extract revealed the presence of tannins, terpenoids, glycosides, and alkaloids, and it exhibited antimicrobial effects [7]. Two dinorditerpenoids, 1,2-dihydroheudelotinol and heudelotinone, and three other compounds, lupeol, *E*-ferullic acid octacosylate, and 3-methylmethylorsellinate, have been isolated from the stem bark and roots of *R. heudelotii* [8]. Seven new tetracyclic triterpenoids ricinodols with potential as 11β-hydroxysteroid dehydrogenase type 1 (11β-HSD1) inhibitors has also been reported to be isolated from *R. heudelotii* [9]. Yakubu et al. [10] also reported the leaf extract to have regulatory effects on artemisinin toxicity when co-administered with artemisinin. This study aims to identify the chemical structure of isolated bioactive compounds in *R. heudelotii* as well as evaluate the compoundsfor in vitro cytotoxic activity against human leukemia (HL-60), human hepatocellular carcinoma (SMMC-7721), human lung adenocarcinoma (A549), human breast cancer cell (MCF-7), and colon adenocarcinoma (SW480) cell lines.

## 2. Results

Nine compounds were isolated from the ethyl acetate fraction of *R. heudelotii* by employing column chromatography, including Sephadex LH-20 and MCI CHP20P gel.

### 2.1. Structural Elucidation of Isolated Compounds

**Figure 1:**
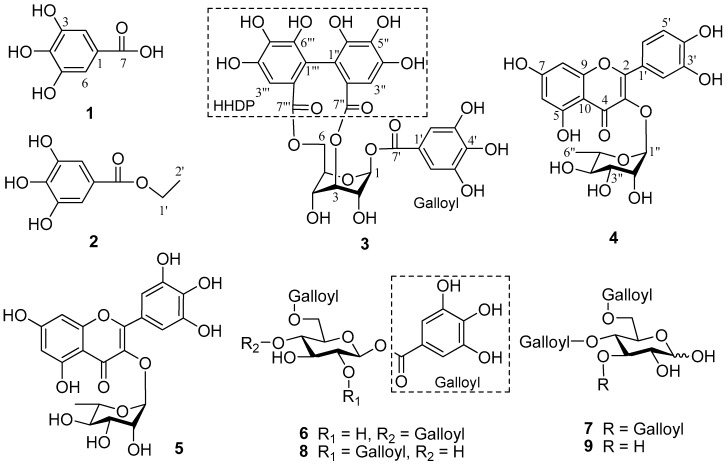
Structures of compounds (**1**–**9**) isolated from *Ricinodendron heudelotii.*

Structure elucidation of the isolated compounds was carried out using spectroscopic techniques: ^1^H- (600 MHz) and ^13^C-NMR (150 MHz) and DEPT along with ^2^D-NMR for some compounds (Figure 1). Compound **1** was obtained as an off-white amorphous powder, showing a molecular ion peak at *m/z* 170 [M]^−^ in the negative ESI-MS. The molecular formula, C_7_H_6_O_5_, was deduced from the combination of ^1^H-, ^13^C-NMR and DEPT spectroscopic data. Compound **1** was identified as gallic acid when the data was compared with those in the literature [11,12]. Compound **2**, an off-white powder, showed a quasi-molecular ion peak at *m/z* 198 [M]^−^ in the negative ESI-MS. Combining with the ^1^H-, ^13^C-NMR and DEPT spectroscopic data, a molecular formula, C_9_H_10_O_5_, was deduced. Comparing the data with those in the literature, compound **2** was identified as gallic acid ethyl ester [13]. Compound **3** was obtained as a yellow amorphous powder, showing a molecular ion peak at *m*/*z* 633 [M]^−^ in the negative ESI-MS. From ^1^H-, ^13^C-NMR, and DEPT spectroscopic data, the molecular formula of C_27_H_22_O_18_ was deduced. Comparing the data with those in the literature, compound **3** was identified as corilagin [14]. Compound **4** was obtained as a yellow amorphous powder. It showed a molecular ion peak at *m/z* 447 [M − H]^−^ in the negative ESI-MS while the molecular formula of C_21_H_20_O_11,_ was deduced from the ^1^H-, ^13^C-NMR, and DEPT spectroscopic data. Likewise, by comparing the data with those in the literature, compound **4** was quercetin-3-*O*-rhamnoside [15,16]. Compound **5** was obtained as a pale yellow amorphous powder showing a quasi-molecular ion peak at *m/z* 463 [M − H]^−^ in the negative ESI-MS, and the molecular formula of C_21_H_20_O_12_, was deduced from ^1^H-, ^13^C-NMR, and DEPT spectroscopic data. By comparing the data with those in the literature, compound **5** was identified as myricetin-3-*O*-rhamnoside [12,17]. Compound **6** was obtained as an off white powder, showing a quasi-molecular ion peak at *m*/*z* 635 [M − H]^−^ in the negative ESI-MS. Combining with the ^1^H-, ^13^C-NMR, and DEPT spectroscopic data, a molecular formula of C_27_H_24_O_18_ was deduced. By comparing the data with those in literature, compound **6** was identified as 1,4,6-tri-*O*-galloyl-β-d-glucose [18]. Compound **7** was obtained as a white amorphous powder. It showed a molecular ion peak at *m*/*z* 636 [M − H]^−^ in the negative ESI-MS. Combining the ^1^H-, ^13^C-NMR, and DEPT spectroscopic data, a molecular formula of C_27_H_24_O_18_ was deduced. The data obtained correlates with those in the literature and compound **7** was identified as 3,4,6-tri-*O*-galloyl-d-glucose [19]. Compound **8**, an off-white amorphous powder with a molecular ion peak at *m/z* 635 [M − H]^−^ in the negative ESI-MS. A molecular formula of C_27_H_24_O_18_, was deduced from the ^1^H-, ^13^C-NMR, and DEPT spectroscopic data. Comparing the data with those in the literature, compound **8** was identified as 1,2,6-tri-*O*-galloyl-β-d-glucose [20,21]. Compound **9** was obtained as a white amorphous powder showing a molecular ion peat at *m*/*z* 483 [M − H]^−^ in the negative ESI-MS. A molecular formula was deduced from the ^1^H-, ^13^C-NMR, and DEPT spectroscopic data. By comparing the data with those in the literature, compound **9** was identified as 4,6-di-*O*-galloyl-d-glucose [22,23].

### 2.2. Spectroscopic Data of Compounds ***1**–**9***

Gallic acid (**1**): Off-white amorphous powder, C_7_H_6_O_5_, negative ESI-MS *m*/*z*: 170 [M]^−^, ^1^H-NMR (600 MHz, acetone-*d*_6_): δ 7.10 (2H, s, H-2,6), ^13^C-NMR (150 MHz, acetone-*d*_6_): 121.0 (C-1), 109.0 (C-2,6), 145.9 (C-3,5), 138.3 (C-4), 168.0 (C-7).

Gallic acid ethyl ester (**2**): Off-white amorphous powder, C_9_H_10_O_5_, negative ESI-MS *m*/*z*: 198 [M]^−^, ^1^H-NMR (600 MHz, acetone-*d*_6_): δ_H_ 0.84 (3H, t, *J* = 7.1 Hz, H-2′), 3.82 (2H, q, *J* = 7.1 Hz, H-1′) 7.05 (2H, s, H-3,5); ^13^C-NMR (150 MHz, acetone-*d*_6_): δ_C_ 120.7 (C-1); 109.4 (C-2,6), 138.0 (C-4), 144.9 (C-3,5), 166.8 (C-7), 60.6 (C-1′), 14.1 (C-2′).

Corilagin (**3**): Off-white amorphous powder, C_27_H_22_O_18_,negative ESI-MS *m*/*z* 633 [M − H]^−^, ^1^H-NMR (600, CD_3_OD): δ_H_ 7.05 (2H, s, H-2′,6′),6.69, 6.65 (each 1H, s, HHDP-3″,3‴), 6.36 (1H, d, *J* = 2.4 Hz, H-1), 4.95 (1H, ddd, *J* = 10.9, 8.0, 1.2 Hz, H-6a), 4.80 (1H, brs, H-3), 4.51 (1H, t, *J* = 9.6 Hz, H-5), 4.46 (1H, dd, *J* = 1.7, 1.2 Hz, H-4), 4.16 (1H, dd, *J* = 8.0, 10.9 Hz, H-6b), 3.98 (1H, brd, *J* = 1.2 Hz, H-2); ^13^C-NMR (150 MHz, CD_3_OD): HHDP δ_C_ 167.1 and 167.6 (C=O), 145.8, 145.1 (C-3″,3‴), 144.9, 144.3 (C-5″,5‴), 136.3, 136.2 (C-4″,4‴), 116.2, 116.1 (C-1″,1‴), 124.1, 123.8 (C-2″,2‴), 107.3, 106.7 (C-6″,6‴); galloyl δ_C_ 165.2 (C=O), 146.2 (C-3′,C-5′), 139.7 (C-4′), 119.4 (C-1′), 109.7 (C-2′,C-6′); glucose δ_C_ 92.9 (C-1), 78.3 (C-3), 76.6 (C-5), 72.4 (C-2), 64.2 (C-6), 62.8 (C-4).

Quercetin-3-*O*-rhamnoside (**4**): Yellow amorphous powder, C_21_H_20_O_11_; negative ESI-MS *m*/*z* 447 [M − H]^−^, ^1^H-NMR (600 MHz, CD_3_OD): δ 7.33 (1H, d, *J* = 2.0 Hz, H-2′), 7.31 (1H, dd, *J* = 2.0, 8.3 Hz, H-6′), 6.91 (1H, d, *J* = 8.3 Hz, H-5′), 6.19 (1H, s, H-6), 6.36 (1H, s, H-8), 5.34 (1H, s, H-1″), 4.22 (1H, brs, H-2″), 3.76 (1H, dd, *J* = 3.2, 9.4 Hz, H-3″), 3.34 (1H, dd, *J* = 7.5, 9.4 Hz, H-4″), 3.41 (1H, m, H-5″), 0.94 (3H, d, *J* = 6.1 Hz, H-6″); ^13^C-NMR (150 MHz, CD_3_OD): δ_C_ 18.7 (C-6″), 71.1 (C-5″), 70.6 (C3″), 71.1 (C-2″), 71.2 (C-4″), 94.3 (C-8), 98.7 (C-6), 102.1 (C-1″), 104.1 (C-10), 115.7 (C-2′), 115.8 (C-5′), 120.8 (C-1′), 122.2 (C-6′), 134.3 (C-3), 145.4 (C-3′), 148.8 (C-4′), 156.7 (C-2), 157.3 (C-9), 161.4 (C-5), 165.6 (C-7), 177.8 (C-4).

Myricetin 3-*O*-rhamnoside (**5**): Yellow amorphous powder, C_21_H_20_O_12_, negative ESI-MS *m*/*z*: 463 [M − H]^−^, ^1^H-NMR (600 MHz, CD_3_OD): δ 6.95 (2H, s, H-2′,6′), 6.36 (1H, d, *J* = 1.6 Hz, H-6), 6.20 (1H, d, *J* = 1.6 Hz, H-8), 5.31 (1H, s, H-1″), 3.80 (1H, dd, *J* = 3.2, 9.5 Hz, H-2″), 3.55 (1H, m, H-3″), 3.17 (1H, m, H-4″), 3.34 (1H, m, H-5″), 0.84 (3H, d, *J* = 6.1 Hz, H-6″). ^13^C-NMR (150 MHz, CD_3_OD): δ_C_ 177.6 (C-4), 163.8 (C-7), 161.4 (C-5), 157.1 (C-9), 156.6 (C-2), 145.9 (C-5′, 3′), 135.4 (C-4′), 135.2 (C-3), 119.7 (C-1′), 107.9 (C-2′), 107.9 (C-6′), 104.3 (C-10), 102.4 (C-1″), 98.8 (C-8), 93.7 (C-6), 71.9 (C-4″), 71.3 (C-5″), 70.8 (C-3″), 70.3 (C-2″), 17.8 (C-6″).

1,4,6-Tri-*O*-galloyl-β-d-glucose (**6**): Off-white amorphous powder, C_27_H_24_O_18_, negative ESI-MS *m*/*z* 635 [M − H]^−^; ^1^H-NMR (600 MHz, CD_3_OD): δ_H_ 7.15, 7.10, 7.06 (each 2H, s, galloyl H-2′,6′), 5.78 (1H, d, *J* = 8.2 Hz, H-1), 5.22 (1H, t, *J* = 9.6 Hz, H-4), 4.45 (1H, dd, *J* = 12.4, 2.0 Hz, H-6a), 4.22 (1H, dd, *J* = 12.4, 4.9 Hz, H-6b), 4.06 (1H, ddd, *J* = 2.0, 4.9, 9.6 Hz, H-5), 3.84 (1H, dd, *J* = 9.2, 9.6 Hz, H-3), 3.65 (1H, dd, *J* = 8.2, 9.2 Hz, H-2).

3,4,6-Tri-*O*-galloyl-d-glucose (**7**): Off-white amorphous powder, C_27_H_24_O_18_, negative ESI-MS *m*/*z* 635 [M − H]^−^, ^1^H-NMR (600 MHz, CD_3_OD): δ_H_ 7.07, 6.99, 6.94 (each 2H, s, galloyl H-2′,6′), 4.42 (1H, m, H-6a), 4.26 (1H, m, H-6b), β-glucose form: δ_H_ 4.76 (1/3H, d, *J* = 7.8 Hz, H-1), 3.56 (1/3H, dd, *J* = 9.5, 7.8 Hz, H-2), 5.45 (1/3H, t, *J* = 9.5 Hz, H-3), 5.35 (1/3H, dd, *J* = 9.5, 9.8 Hz, H-4), 4.07 (1/3H, m, H-5), α-glucose form: δ_H_ 5.25 (2/3H, d, *J* = 3.6 Hz, H-1), 3.81 (2/3H, dd, *J* = 9.8, 3.6 Hz, H-2), 5.68 (2/3H, t, *J* = 9.8 Hz, H-3), 5.36 (2/3H, t, *J* = 9.8 Hz, H-4), 4.47 (2/3H, m, H-5).

1,2,6-Tri-*O*-galloyl-β-d-glucose (**8**): Off-white amorphous powder, C_27_H_24_O_18_, negative ESI-MS: *m*/*z* 635 [M − H]^−^, ^1^H-NMR (600 MHz, CD_3_OD): δ_H_ 7.10, 7.04, 7.00 (each 2H, s, galloyl H-2′,6′), 5.92 (1H, d, *J* = 8.5 Hz, H-1), 5.23 (1H, dd, *J* = 8.5, 9.6 Hz, H-2), 4.56 (1H, dd, *J* = 2.0, 12.2 Hz, H-6a), 4.47 (1H, dd, *J* = 4.8, 12.2 Hz, H-6b), 3.83 (1H, ddd, *J* = 2.0, 4.8, 9.6 Hz, H-5), 3.81 (1H, t, *J* = 9.6 Hz, H-3), 3.66 (1H, t, *J* = 9.6 Hz, H-4); ^13^C-NMR (150 MHz, CD_3_OD): δ_C_ 63.1 (C-6), 69.6 (C-4), 72.7 (C-5), 74.4 (C-3), 74.2 (C-2), 93.2 (C-1), 117.0, 119.1, 119.2 (galloyl C-1′), 109.2, 108.8, 108.8 (galloyl C-2′,6′), 145.3, 145.4, 145.8 (galloyl C-3′,5′), 138.6, 138.7, 139.1 (galloyl C-4′), 164.1, 164.9, 165.7 (C=O).

4,6-Di-*O*-galloyl-β-d-glucose (**9**): Off-white amorphous powder, C_20_H_20_O_14_, negative ESI-MS: *m*/*z* 483 [M − H]^−^, ^1^H-NMR (600 MHz, CD_3_OD): δ_H_ 7.09, 7.07, 7.08, 7.06 (each 1H, s, 2× galloyl H-2′,6′), 5.13 (1H, t, *J* = 9.4 Hz, H-4), 4.39 (1H, m, H-6a), 4.18 (1H, m, H-6b), α-glucose form: 5.17 (1/2H, d, *J* = 3.7 Hz, H-1), 3.98 (1/2H, t, *J* = 9.4 Hz, H-3), 4.53 (1/2H, dd, *J* = 3.7, 9.6 Hz, H-2), β-glucose form: δ_H_ 4.60 (1/2H, d, *J* = 7.8 Hz, H-1), 3.89 (1/2H, m, H-5), 3.70 (1H, t, *J* = 9.3 Hz, H-3), 3.31 (1/2H, dd, *J* = 9.3, 7.8 Hz, H-2).

### 2.3. Bioassay

In an in vitro cytotoxicity assay, compounds **1**–**5** were applied to HL-60, SMMC-7721, A-549, MCF-7, and SW-480 cancer cells to assess their cytotoxic effects. Compound **3** (Corilagin) exhibited the most cytotoxic activity against MCF-7 cells with IC_50_ values of 33.18 and 25.81 µg/mL for HL-60 cells. The IC_50_ value of compound **3** in MCF-7 cells is comparable with the positive control drug (cisplatin), having an IC_50_ value of 27.43 (Table 1). Corilagin had 29.19, 4.91, and 49.82% inhibition for A-549, SMMC-7721, and SW-480 respectively. The inhibition rate of other compounds (gallic acid, gallic acid ethyl ester, quercetin-3-*O*-rhamnoside, myricetin-3-*O*-rhamnoside) wasless than 50% at the concentration of 40 µM (Table 2), suggesting that they showed no significant inhibitory activity against the cancer cell lines; therefore, their IC_50_ values were not measured.

**Figure 2:**
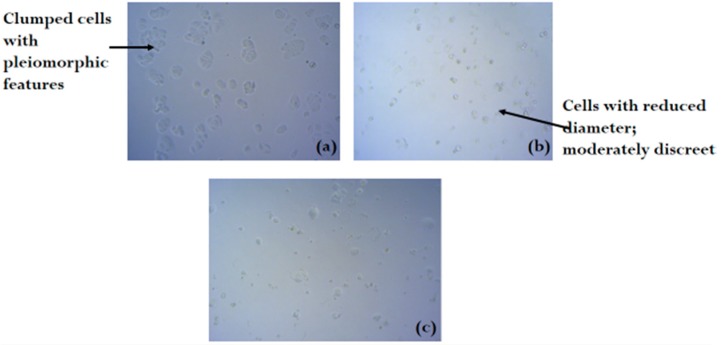
Photomicrograph of breast cancer cell line (**a**) untreated, (**b**) treated with cisplatin, and (**c**) treated with compound **3** (H&E × 100).

**Figure 3:**
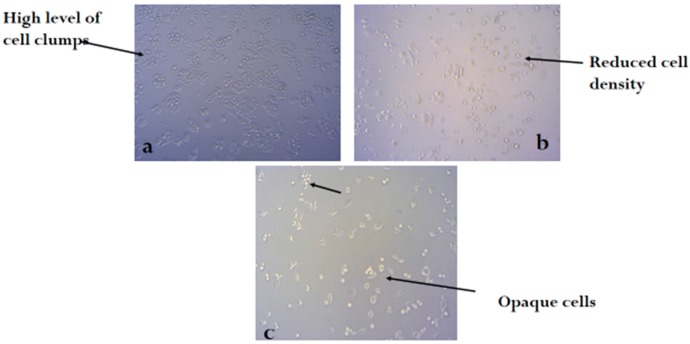
Photomicrograph of colon cancer cell line (**a**) untreated, (**b**) treated with cisplatin, and (**c**) treated with compound **3** (H&E × 100).

**Figure 4:**
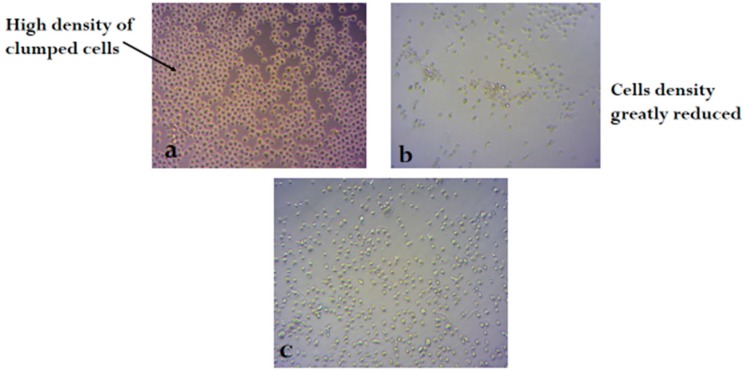
Photomicrograph of leukemia cancer cell line (**a**) untreated, (**b**) treated with cisplatin, and (**c**) treated with compound **3** (H&E × 200).

Figure 2, Figure 3 and Figure 4 shows the photomicrograph of the cytotoxic activities of compound **3** on breast, colon, and leukemia cancer cell lines with relatively similar activity when compared with cisplatin (control group).

## 3. Discussion

After many years of research on cancer, cancer still remains a destructive disease responsible for over one quarter of the deaths recorded globally. It is one of the major health obstacles, representing the second largest cause of mortality [24]. This is mainly because cancer chemotherapy is often mitigated by a high degree of toxicity to normal cells or failure of treatment due to the development of resistance to anticancer drugs. However, medicinal plants have been increasingly shown to possess anticancer properties, and this has recently become the subject of some scientific studies [25] because they are considered to be less toxic and can be effective for treatment of different diseases. To date, the therapeutic pre-clinical screening of medicinal plants against multiple cancer cells and characterization of the bioactive components in these plants are limited. Our study reports a bioactive compound with cytotoxicity effects in the leaf extracts of *R. heudelotii* and its potential to be developed for use for cancer treatment.

Corilagin (compound **3**) was isolated and structurally identified from the ethyl acetate fraction of the ethanolic extract of *R. heudelotii* leaves in our study. Only compound **3** showed significant and comparable cytotoxic property relative to cisplatin. Corilagin has been previously identified in several other plants, especially those of the Euphorbiaceae family. It was firstly isolated from *Caesalpinia coriaria* in 1951, hence the name of the molecule [26]. It was later identified as a weak carbonic anhydrase inhibitor [27]. Studies have reported its antibacterial, hepatoprotective, anti-oxidant [28], anti-inflammatory [29], anti-hypertensive [30], and anti-tumor activities [31]. Yang et al. [32] reported that corilagin can block glioblastoma cells and stem-like cells’ proliferation. In this study, corilagin was found to exhibit an inhibitory effect on HL-60, MCF-7, and SW480 cell growth by 76.23, 57.93, and 49.82%, respectively (Table 2). This could be linked to its ability to activate the Jun N-terminal kinase (JNK) signaling pathway [33]. When these pathways are turned on, it can lead to the activation of the proapoptotic β-cell lymphoma 2 (Bcl-2) protein through phosphorylation and/or upregulation of gene expression and hence mitochondrial cell death [34]. Breast cancer cells treated with corilagin showed reduced cell diameters when compared to the untreated cells, implying that the compound caused cell death of most of the cancerous cells (Plate 1). Corilagin is an ellagitannin, which have been reported to induce pro-apoptotic effects in in both MDA-MB-231 and MCF-7 breast cancer cells [35]. Kuo et al. [36] observed that ellagitannins from *Terminalia arjuna* bark extract induced apoptosis in A549 cells and in human breast adenocarcinoma MCF-7 cells via blockage of cell-cycle progression in the G0/G1 phase. The cytotoxic properties of tannins are characterised by the release of reactive oxygen species (ROS), which is followed by a reduction in the thioredoxin, superoxide dismutase, and in the level of redox active proteins [37]; this might explain the cytotoxic activity of compound **3**. The chemopreventive activity of tannins, especially the ellagitannins and hydrolysable tannins, has been linked to activation of apoptosis [38]. According to the findings of Jia et al. [31], corilagin inhibited SKOv3ip by inducing cell cycle arrest and enhancing apoptosis in the cancer cell lines. Down regulation of cyclin B1 and phosphor-*cdc 2* after corilagin treatment on cancer cells was discovered [31]. Corilagin has also been reported to cause the release of TNF-α inhibitor in inflammation [39], decrease the secretion of the transforming growth factor beta 1 (TGF-β1) in a dose dependent manner [31], and inhibit hepatitis C viral replication in vitro [40]. Corilagin enhanced apoptosis by inducing cell cycle arrest at the G2/M stage in ovarian cancer cells. Anti-cancer strategies have focused on possible ways of deactivating the G2/M checkpoint, thereby causing cancer cells to undergo mitosis, and leading to increased DNA damage and then cell death [41,42]. Ming et al. [43] also reported the inhibitory action of corilagin on hepatocellular carcinoma cells (HCC) at the G2/M phase by upregulating p53 and p21, and downregulating cyclin B1, *cdc 2*, and pAKT. Selectivity index (SI) signifies the differential activity of compounds; the greater the SI value, the more selective the compound [44]. The SI data, as shown in Table 3, indicate that corilagin was non-selective to HL-60, MCF-7, and SW480 cancer cell lines. This suggests its general toxicity to the cell. Adverse effects caused by treatment and increased resistance to tumor cells, leading to treatment failure, has led to a search for therapeutic alternatives. Therefore, it is important to find selective bioactive compounds that could arrest tumor cell proliferation.

## 4. Materials and Methods

### 4.1. General Experimental Procedures

^1^D- and ^2^D-NMR spectra were recorded in CD_3_OD and acetone-*d*_6_ with Bruker Avance III600 spectrometer (Bruker, Fällande, Switzerland) operating at 600 MHz for ^1^H-NMR, and 150 MHz for ^13^C-NMR, respectively. Coupling constants are expressed in hertz (Hz), and chemical shifts are given on a δ (parts per million, ppm) scale with tetramethylsilane (TMS) as an internal standard. ESI mass spectra were recorded on a VG Auto Spec-300 spectrometer (Waters, Milford, MA, USA). Column chromatography (CC) was performed on Sephadex LH-20 (25–100 μm, GE Healthcare Bio-Science AB, Uppsala, Sweden), MCI-gel CHP20P (75–150 μm, Mitsubishi Chemical Co, Ltd., Tokyo, Japan), and Toyopearl HW-40F (Tosoh Co. Ltd., Tokyo, Japan). Thin-layer chromatography (TLC) was performed on silica gel H-precoated plates, 0.2–0.25 mm thick (Qingdao Haiyang Chemical Co., Qingdao, China), with benzene–ethylformate–formic acid (3:6:1 or 2:7:1, *v*/*v*), and compounds were detected by spraying with 2% ethanolic FeCl_3_ and 10% sulfuric ethanol solution followed by heating.

### 4.2. Plant Sample Collection

*Ricinodendron heudelotii* leaves were collected within Covenant University premises Ota, Ogun, Nigeria. The plant was identified by Dr. J.O. Popoola (Botanist, Biological Science Department, Covenant University, Ota, Ogun State, Nigeria) and a voucher specimen was prepared and submitted in the Forest research Institute of Nigeria, Ibadan with voucher number, FHI 110573. Leaves were dried at room temperature (25 °C) and blended using an electric blender into a coarse powder.

### 4.3. Extraction and Solvent Partitioning ofthe Crude Extract

The powdered leaf samples (10 kg) were extracted using 95% ethanol by macerationat room temperature, the filtrate was further condensed under reduced temperature and pressure, and the yield of the extract obtained was 11.75%. The concentrated crude extract (1 kg) was suspended in 1 L distilled water and partitioned in sequence with petroleum ether (Pet; 8 L), ethyl acetate (EtOAc; 8 L), and *n*-butanol (8 L). The solvent fractions were concentrated to dryness to afford four (4) fractions as: Pet, EtOAc, *n*-butanol, and aqueous fractions. The EtOAc fraction was selected for subsequent isolation procedures.

### 4.4. Isolation of Compounds fromthe Partitioned Fraction

The ethylacetate fraction (246 g) was loaded into a column containing Sephadex LH-20 (800 g). The eluent was a mixture of water and methanol in different ratios starting with 100:0 to 0:100. Fractions with similar TLC patterns were combined to afford 13 fractions. Fraction 11 (36 g) was chromatographed on a Sephadex LH-20 column and MCI-gel CHP20P eluting with water:methanol (4:1) to afford compounds **1** (8.53 mg), **2** (2.1 mg), and **3** (12 mg). Fraction 12 (3.8 g) was subjected to repeated column chromatography also eluting with H_2_O and methanol (60:1) to afford compound **4** (98 mg) and **5** (13.2 mg). Compounds **6** (9.9 mg), **7** (2.6 mg), **8** (2.7 mg), and **9** (4.4 mg) were obtained from fraction 13 (14.5 g) after repeated purification by chromatography on a Sephadex LH-20, MCI-gel CHP20P and Toyopearl HW-40F columns eluting with H_2_O/methanol.

### 4.5. Bioassay

#### 4.5.1. Cell Culture

Cancer cell lines, HL-60, SMMC-7721, A-549, MCF-7, and SW-480, were obtained from the American Type Culture Collection Center (ATCC) (Manassas, VA, USA). They were cultured in RMPI-1640 or DMEM medium supplemented with 10% bovine serum albumin (Gibco, Grand Island, NY, USA). Prior to exposure to drugs, the cancer cell lines were cultured in a CO_2_ incubator for 48 h and the cell density was adjusted to 5 × 10^4^ cells/well.

#### 4.5.2. Sample Preparation

Compounds (1 mg/mL) were dissolved in either DMSO or H_2_O depending on their solubility. Samples (2 µL) were suspended in centrifuge tubes containing 48 µL of complete medium to make a final volume of 100 µL.

#### 4.5.3. Assay Procedures

The cytotoxicity assay was evaluated using the 3-(4,5-dimethylthiazol-2-yl)-5-(3-carboxymethoxyphenyl)-2-(4-sulfophenyl)-2*H*-tetrazolium, inner salt (MTS) method by measuring the absorbance of formazan precipitate formed in the cells based on MTS reduction [45]. In this assay, cells were seeded into each of the wells of 96-well cell culture plates, incubated for 12h at 37 °C, and then the test compounds (40 µM) were added and allowed to incubate for 48 h. Each cell line was exposed to different concentrations (0.064, 0.32, 1.6, 8, and 40 µM) of the test compounds in triplicates using cisplatin-an anticancer drug as the positive control. The absorbance of the lysate was measured using a 96-well micro plate reader (Bio-Rad 680, Hercules, CA, USA) at 490 nm. Compounds with a growth inhibition rate of 50% were further evaluated at concentrations of 0.064, 0.32, 1.6, 8, and 40 µM in triplicates with cisplatin as positive control. The concentration that inhibited 50% of the cell growth were expressed as IC_50_, which was calculated using Reed and Muench’s method [46,47]. The percentage inhibition (I%) was calculated as follows:

Inhibition (%) = (Cc − Cs)/Cc × 100(1)

where Cc = viable cell count of negative control, Cs = viable cell count of sample, IC_50_ was calculated by the following equation:

Log_10_ (IC_50_) = (Log_10_ (C_L_) (I_H_ − 50) + log_10_ (C_H_) (50 − I_L_))/(I_H_ − I_L_) (2)

IC_50_ = 10 ^Log^_10_^(IC^_50_^)^; I_H_: I% above 50%; I_L_: I% below 50%; C_H_: High drug concentration; and C_L_: Low drug concentration.

### 4.6. Selectivity Index

The selectivity index (SI) for the cytotoxicity of corilagin was calculated using the ratio of the IC_50_ value of the compound on a normal cell line to the IC_50_ value of the compound on cancer cell lines [48].

### 4.7. Statistical Analysis

Samples are expressed as mean ± standard deviation or standard error of mean.

## 5. Conclusions

In conclusion, we report significant cytotoxic activities of corilagin isolated from leaf extract of *R. heudelotii* on a breast cancer cell line, and its potential for development as an anticancer agent to reduce the burden of cancer diseases globally.

## Figures and Tables

**Table 1:** IC_50_ values (µg/mL) of corilagin (**3**) isolated from *R. heudelotii* against some cancer cell lines.

Sample	HL-60	MCF-7	SW480	BEAS-2B
Corilagin (**3**)	25.81 ± 0.67 *	33.18 ± 0.76 *	37.04 ± 1.06 *	16.32 ± 1.43
Cisplatin	2.32 ± 0.07	27.43 ± 1.26	10.19 ± 1.03	---

* Significant (*p* < 0.05) when compared to the control (Cisplatin).

**Table 2:** Percentage of inhibition (I%) of compounds **1**–**5** (40 µM) isolated from *R. heudelotii* on different cell lines.

Compounds	HL-60	A-549	SMMC-7721	MCF-7	SW480
**1**	14.80 ± 5.00	13.67 ± 2.91	5.85 ± 1.16	18.41 ± 4.00	10.04 ± 3.51
**2**	12.26 ± 2.29	11.82 ± 0.14	5.19 ± 0.83	24.76 ± 3.30	1.15 ± 0.28
**3**	76.23 ± 0.51	29.19 ± 1.42	4.91 ± 1.43	57.93 ± 3.19	49.82 ± 3.80
**4**	4.56 ± 2.61	5.49 ± 2.01	1.12 ± 1.05	14.95 ± 0.82	2.44 ± 2.49
**5**	0.65 ± 2.87	8.32 ± 3.46	1.62 ± 1.05	10.67 ± 4.05	12.45 ± 3.66

**Table 3:** Selectivity index of compound **3** (corilagin).

Sample	HL-60	MCF-7	SW480
Corilagin (**3**)	0.63	0.49	0.44

## References

[1] Sylla B.S., Wild C.P. (2012). A million Africans a year dying from cancer by 2030: What can cancer research and control offer to the continent?. Int. J. Cancer.

[2] Torre L.A., Bray F., Siegel R.L., Ferlay J., Lortet-Tieulent J., Jemal A. (2012). Global Cancer Statistics, 2012. Cancer J. Clin..

[3] Hanahan D., Weinberg R.A. (2011). Hallmarks of cancer: The next generation. Cell.

[4] López-Abente G., Aragonés N., Pérez-Gómez B., Pollan M., García-Pérez J., Ramis R., Fernández-Navarro P. (2014). Time trends in municipal distribution patterns of cancer mortality in Spain. BMC Cancer.

[5] Cragg G.M., Newman D.J., Snader K.M. (1997). Natural products in drug discovery and development. J. Nat. Prod..

[6] Momeni J., Djoulde R.D., Akam M.T., Kimbu S.F. (2005). Chemical constituents and antibacterial activities of the stem bark extracts of *Ricinodendron heudelotii* (Euphorbiaceae). Indian J. Pharm. Sci..

[7] Yakubu O.F., Adebayo A.H., Famakinwa T.O., Adegbite O.S., Ishola T.A., Imonikhe L.O., Adeyemi O.A., Awotoye O.A., Iweala E.E.J. (2018). Antimicrobial and toxicological studies of *Ricinodendron heudelotii* (Baill.). Asian J. Pharm. Clin. Res..

[8] Kimbu S.F., Keumedjio F., Sondengam L.B., Connolly J.D. (1991). Two dinorditerpenoids from *Ricinodendron heudelotii*. Phytochemistry.

[9] Yu J.H., Shen Y., Wu Y., Leng Y., Zhang H., Yue J.M. (2015). Ricinodols A—G: New tetracyclic triterpenoids as 11β-HSD1 inhibitors from *Ricinodendron heudelotii*. RSC Adv..

[10] Yakubu O.F., Adebayo A.H., Okechukwu E.S., Adeyemi O.A., Iweala E.J., Zhang Y. (2018). Co-administration of artemisinin and *Ricinodendron heudelotii* leaf extract—Effects on selected antioxidants and liver parameters in male Wistar rats. Comp. Clin. Path..

[11] Eldahshan O.A. (2011). Isolation and structure elucidation of phenolic compounds of carob leaves grown in Egypt. J. Biol. Sci..

[12] Soro Y., Kassi A.B.B., Bamba F., Siaka S., Toure S.A., Coustard J.M. (2012). Flavonons and gallic acid from leaves of *Santaloides afzelii* (Connaraceae). Rasayan J. Chem..

[13] Mori T., Chang C., Maurtua D., Hammond G.B. (2006). Isolation of the active compound in *Mauria heterophylla*, a peruvian plant with antibacterial activity. Phytother. Res..

[14] Zheng Z., Chen L., Liu S., Deng Y., Zheng G., Gu Y., Ming Y. (2016). Bioguided fraction and isolation of the antitumor components from *Phyllanthus niruri* L.. BioMed Res. Int..

[15] Bose S., Maji S., Chakraborty P. (2013). Quercitrin from *Ixora coccinea* leaves and its anti-oxidant activity. J. Pharmacol. Sci. Technol..

[16] Ibrahim M.T., Abu S.A., Sleem A.A. (2017). Phytochemical and biological investigation of *Thunbergia grandiflora*. J. Pharmacol. Phytochem..

[17] Zhong X.N., Otsuka H., Ide T., Hirata E., Takushi A., Takeda Y. (1997). Three flavonol glycosides from leaves of *Myrsine seguinii*. Phytochemical.

[18] Hassan M., Kubmarawaı D., Oladosu P. (2017). Antiallergic polyphenols from *Citharexylum spinosum*. Trends Phytochem. Res..

[19] Si C.L., Xu J., Lu Y.Y., Su Z., Su Y.F., Bae Y.S. (2011). Hydrolysable tannins from *Juglans sigillata* stem barks. Biochem. Syst. Ecol..

[20] Bag A., Bhattacharyya S.K., Chattopadhyay R.R. (2013). Isolation and identification of a gallotannin 1,2,6-tri-*O*-galloyl-β-*d*-glucopyranose from hydroalcoholic extract of *Terminaliachebula* fruits effective against multidrug-resistant uropathogens. J. Appl. Microbiol..

[21] Nawwar M.A.M., Hussein S.A.M., Merfort I. (1994). NMR spectral analysis of polyphenols from *Punica granatum*. Phytochemical.

[22] Okuda T., Ito H. (2011). Tannins of constant structure in medicinal and food plants-hydrolyzable tannins and polyphenols related to tannins. Molecules.

[23] Saijo R., Nonaka G., Nishioka I. (1989). Tannins and related compounds. LXXXIV. Isolation and characterization of five new hydrolyzable tannins from the bark of *Mallotus japonicus*. Chem. Pharm. Bull..

[24] Rang H.P. (2003). Pharmacology.

[25] Abd-Rabou A.A., Zoheir K.M.A., Ahmed H.H. (2012). Potential impact of curcumin and taurine on human hepatoma cells using Huh-7 cell line. Clin. Biochem..

[26] Schmidt O.T., Lademann R. (1951). Corilagin, ein weiterer kristallisierter Gerbstoff aus Dividivi. X. Mitteilung über natürliche Gerbstoffe. Justus Liebigs Annalen Der. Chemie.

[27] Satomi H., Umemura K., Ueno A., Hatano T., Okuda T., Noro T. (1993). Carbonic anhydrase inhibitors from the pericarps of *Punica granatum* L.. Biol. Pharm. Bull..

[28] Kinoshita S., Inoue Y., Nakama S., Ichiba T., Aniya Y. (2007). Antioxidant and hepatoprotective actions of medicinal herb, *Terminalia catappa* L. from Okinawa Island and its tannin corilagin. Phytomedicine.

[29] Li H.R., Liu J., Zhang S.L., Luo T., Wu F., Dong J.H., Guo Y.J., Zhao L. (2017). Corilagin ameliorates the extreme inflammatory status in sepsis through TLR4 signaling pathways. BMC Complement. Altern. Med..

[30] Cheng J.T., Lin T.C., Hsu F.L. (1995). Antihypertensive effect of corilagin in the rat. Can. J. Physiol. Pharmacol..

[31] Jia L., Jin H., Zhou J., Chen L., Lu Y., Ming Y., Yu Y. (2013). A potential anti-tumor herbal medicine, Corilagin, inhibits ovarian cancer cell growth through blocking the TGF-β signaling pathways. BMC Complement. Altern. Med..

[32] Yang W.T., Li G.H., Li Z.Y., Feng S., Liu X.Q., Han G.K., Zhang H., Qin X., Zhang R., Nie Q. (2016). Effect of corilagin on the proliferation and NF-κB in U251 glioblastoma cells and U251 glioblastoma stem-like cells. Evid. Based Complement. Altern. Med..

[33] Kim S.J., Park K.M., Kim N., Yeom Y.L. (2006). Doxorubicin prevents endoplasmic reticulum stress-induced apoptosis. Biochem. Biophys. Res. Commun..

[34] Puthalakath H., O’Reilly L.A., Gunn P., Lee L., Kelly P.N., Huntington N.D., Hughes P.D., Michalak E.M., MicKimm-Breschkin J., Motoyama N. (2007). ER stress triggers apoptosis by activating BH3-only protein bim. Cell.

[35] Ismail T., Calcabrini C., Diaz A.R., Fimognari C., Turrini E., Catanzaro E., Akhtar S., Sestili P. (2016). Ellagitannins in cancer chemoprevention and therapy. Toxins.

[36] Kuo P.L., Hsu Y.L., Lin T.C., Lin L.T., Chang J.K., Lin C.C. (2005). Casuarinin from the bark of *Terminalia arjuna* induces apoptosis and cell cycle arrest in human breast adenocarcinoma MCF-7 cells. Planta Med..

[37] Naus P.J., Henson R., Bleeker G., Wehbe H., Meng F., Patel T. (2007). Tannic acid synergizes the cytotoxicity of chemotherapeutic drugs in human cholangiocarcinoma by modulating drug efflux pathways. J. Hepatol..

[38] Le V., Esposito D., Grace M.H., Ha D., Pham A., Bortolazzo A., Bevens Z., Kim J., Komarnytsky S., Lila M.A. (2014). Cytotoxic effects of ellagitannins isolated from walnuts in human cancer cells. Nutr. Cancer.

[39] Gambari R., Borgatti M., Lampronti I., Fabbri E., Brognara E., Bianchi N., Piccagli L., Yuen M.C.W., Kan C.W., Hau D.K. (2012). Corilagin is a potent inhibitor of NF-κB activity and downregulates TNF-alpha induced expression of IL-8 gene in cystic fibrosis IB3-1 cells. Int. Immunopharmacol..

[40] Reddy B.U., Mullick R., Kumar A., Sudha G., Srinivasan N., Das S. (2014). Small molecule inhibitors of HCV replication from Pomegranate. Sci. Rep..

[41] Singh R.P., Dhanalakshmi S., Agarwal R. (2002). Phytochemicals as cell cycle modulators a less toxic approach in halting human cancers. Cell Cycle.

[42] Thakur V.S., Deb G., Babcook M.A., Gupta S. (2014). Plant phytochemicals as epigenetic modulators: Role in cancer chemoprevention. AAPS J..

[43] Ming Y., Zheng Z., Chen L., Zheng G., Liu S., Yu Y., Tong Q. (2013). Corilagin inhibits hepatocellular carcinoma cell proliferation by inducing G2/M phase arrest. Cell Biol. Int..

[44] Peña-Morán O.A., Villarreal M.L., Álvarez-Berber L., Meneses-Acosta A., Rodríguez-López V. (2016). Cytotoxicity, post-treatment recovery, and selectivity analysis of naturally occurring podophyllotoxins from *Bursera fagaroides* var. *fagaroides* on Breast cancer cell lines. Molecules.

[45] Iweala E.E.J., Liu F.F., Cheng R.R., Li Y., Omonhinmin C.A., Zhang Y.J. (2015). Anti-cancer and free radical scavenging activity of some Nigerian food plants in vitro. Int. J. Cancer Res..

[46] Reed L.J., Muench H. (1938). A simple method of estimating fifty percent endpoints. Am. J. Hyg..

[47] Adebayo A.H., Tan N.H., Akindahunsi A.A., Zeng G.Z., Zhang Y.M. (2010). Anticancer and antiradical scavenging activity of *Ageratum conyzoides* L. (Asteraceae). Pharmacogn. Mag..

[48] Dahham S.S., Tabana Y.M., Iqbal M.A., Ahamed M.B.K., Ezzat M.O., Majid A.S.A., Majid A.M.S.A. (2015). The anticancer, antioxidant and antimicrobial properties of the sesquiterpene β-Caryophyllene from the essential oil of *Aquilaria crassna*. Molecules.

